# Gene expression in human fungal pathogen *Coccidioides immitis* changes as arthroconidia differentiate into spherules and mature

**DOI:** 10.1186/1471-2180-13-121

**Published:** 2013-05-28

**Authors:** Suganya Viriyakosol, Akul Singhania, Joshua Fierer, Jonathan Goldberg, Theo N Kirkland, Christopher H Woelk

**Affiliations:** 1 Department of Medicine, University of California San Diego, La Jolla, CA 92093, USA; 2 Research Service, Veterans Affairs San Diego Healthcare System(111F), 3350 La Jolla Village Dr, San Diego, CA 92161, USA; 3 Department of Pathology, University of California San Diego, La Jolla, CA 92093, USA; 4 The Broad Institute, Cambridge, MA 02142, USA

**Keywords:** Coccidioides, Fungus, Spherules, Mycelia, Gene expression, Transcriptome

## Abstract

**Background:**

*Coccidioides immitis* is a dimorphic fungus that causes disease in mammals, including human beings. It grows as a mycelium containing arthroconidia in the soil and in the host arthroconidia differentiates into a unique structure called a spherule. We used a custom open reading frame oligonucleotide microarray to compare the transcriptome of *C. immitis* mycelia with early (day 2) and late stage (day 8) spherules grown *in vitro*. All hybridizations were done in quadruplicate and stringent criteria were used to identify significantly differentially expressed genes.

**Results:**

22% of *C. immitis* genes were differentially expressed in either day 2 or day 8 spherules compared to mycelia, and about 12% of genes were differentially expressed comparing the two spherule time points. Oxireductases, including an extracellular superoxide dismutase, were upregulated in spherules and they may be important for defense against oxidative stress. Many signal transduction molecules, including pleckstrin domain proteins, protein kinases and transcription factors were downregulated in day 2 spherules. Several genes involved in sulfur metabolism were downregulated in day 8 spherules compared to day 2 spherules. Transcription of amylase and α (1,3) glucan synthase was upregulated in spherules; these genes have been found to be important for differentiation to yeast in *Histoplasma*. There were two homologs of 4-hydroxyphenylpyruvate dioxygenase (*4-HPPD*); transcription of one was up- and the other downregulated. We tested the effect of a *4-HPPD* inhibitor, nitisinone, on mycelial and spherule growth and found that it inhibited mycelial but not spherule growth.

**Conclusions:**

Transcription of many genes was differentially expressed in the process of arthroconidia to spherule conversion and spherule maturation, as would be expected given the magnitude of the morphologic change. The transcription profile of early stage (day 2) spherules was different than late stage (day 8) endosporulating spherules. In addition, very few genes that are important for spore to yeast conversion in other dimorphic fungi are differentially expressed in *C. immitis* mycelia and spherules suggesting that dimorphic fungi may have evolved different mechanisms to differentiate from mycelia to tissue invasive forms.

## Background

There are two closely related species of *Coccidioides*: *immitis* and *posadasii* that are found in different geographic areas [1]. They belong to the order Onygenales and are members of the phylum *Ascomycota*. Both *Coccidioides* species are indigenous to the New World where they grow as molds in the alkaline desert soils, primarily in North America, but also in scattered desert areas in South America [2]. These organisms are pathogenic for mammals (including humans), and it is estimated that there are ~150,000 infections annually in the US, primarily in the southwestern region [3]. The soil form of these fungi is a mold that produces infectious spores (arthroconidia) that can become airborne if the soil is disturbed. Arthroconidia are ~ 4 micron in diameter and when inhaled into the lung they can cause pneumonia. Inside the host, under the influence of temperature and partial pressure of CO_2_, the organism undergoes a remarkable transformation into spherules, the pathognomonic tissue form of *Coccidioides.* Arthroconidia first round up and then they start to enlarge and transform. As they grow their cytoplasm undergoes internal segmentation to produce hundreds of endospores that are released when a spherule ruptures [4,5]. These endospores in turn enlarge into spherules and replication continues until the host immune response controls the process or the host dies. The two species of *Coccidioides* have the same life cycle and there is no known difference in the clinical disease caused by infection with the two species.

The natural history of coccidioidomycosis is very variable. About 60% of infections are mild and go undiagnosed, but in Arizona (a hyper-endemic region) coccidioidomycosis is a leading cause of symptomatic pneumonia [6]. Most of those infections resolve spontaneously but they can leave residual solitary granulomas or occasionally thin-walled cavities. In about 5% of cases infection does not remain confined to the lung and spreads to extra-pulmonary sites. This spread can be an overwhelming, life threatening process, or it can manifest as isolated skin, bone, joint, or meningeal infections. The last is uniformly fatal without treatment. Most people with dissemination suffer from prolonged and debilitating infections that are difficult to treat [7]. People who are immunosuppressed, either by disease or iatrogenically, are at high risk for dissemination but the majority of disseminated cases occur in previously healthy individuals with no known immunological defects [8].

As with all the dimorphic pathogenic fungi (*Blastomyces dermatitidis*, *Histoplasma capsulatum, Paracoccidioides brasiliensis* and *Sporothrix schenckii)* the pathogenic form in tissue looks completely different form the saprobic mycelial form found in the environment. In coccidioidomycosis spherule formation is required for pathogenicity [9], as exemplified by two mutant strains [10,11]. Both of these mutants fail to form spherules and although mice can be infected, the infection resolves spontaneously.

One fundamental but poorly understood issue that is relevant to all the dimorphic pathogenic fungi is how they differentiate from a mold (*i.e.,* arthroconidia in mycelia) to the pathogenic form (*i.e.,* spherules). It is possible to induce spherule formation *in vitro* by incubating arthroconidia at an elevated temperature (42°C) in a 14% CO_2_ atmosphere in a medium designed to promote the growth of spherules (Converse media) [12]. We chose to study gene expression in early spherules (day 2 in culture) that have not yet begun to form endospores and late spherules (day 8 in culture) that have formed endospores. The development of early and late spherules has been described [4,5]. *C. immitis* spherules do not rupture and release endospores when cultured in Converse media in our hands. We chose to compare gene expression in early and late spherules to mycelial gene expression to see whether gene expression varied as the spherules matured. We analyzed gene expression using a custom *C. immitis* oligonucleotide array platform constructed to probe the expression of every known and predicted open reading frame (ORF). Our hypothesis was that a large fraction of the genome would be differentially expressed in spherules compared to mycelia. We also hypothesized that many of the genes that are known to be important for mycelial to yeast conversion in other dimorphic pathogenic fungi would also be differentially expressed in the transition to spherules. Microarray gene expression analysis identified a large number of genes differentially expressed between the mycelial and spherule forms of the pathogen. The protein families (PFAM) and gene ontology (GO) terms significantly over-represented in the sets of differentially expressed genes were identified in order to better understand the higher biological processes being affected. Many genes associated with such families or terms were confirmed by real-time quantitative PCR (RT-qPCR). A study of *C. immitis* gene expression by Whiston et al. using RNA-Seq comparing transcript differences between mycelia and day 4 spherules was recently published and allowed us to compare our results to their results obtained at a time point intermediate in spherule development [13].

## Methods

### Growth of mycelia and spherules

*C. immitis* RS strain directly isolated from infected mice was grown on Mycosel agar (3.6% Mycosel agar, 0.5% yeast extract, and 50 μg/ml gentamicin). The animal protocol for infecting mice was approved by the Subcommittee on Animal Studies #07-017. The plates were incubated at 30°C until the mycelia covered the surface of the agar. Arthroconidia were harvested from the plate after 6 weeks of incubation at 25°C by adding 25 ml of saline. The plate was gently scraped using cell scraper and the fluid transferred to a 50 ml tube that was then vigorously mixed for 10 sec and centrifuged at 3000 rpm for 10 min at 4°C. The supernatant containing floating mycelia was discarded. The pellet containing arthroconidia was re-suspended in 10 ml saline and the suspension was passed through a 40 μM nylon cell strainer (BD Falcon). The strained suspension was centrifuged again and the pellet used to produce mycelia and spherules.

To grow mycelia, arthroconidia were washed 2 times with glucose-yeast extract (GYE) media and 2×10^6^ spores/ml were incubated in 250 ml flat-bottom Erlenmeyer flasks (Corning) in 50 ml GYE media. Four flasks were cultured in a 30°C incubator without shaking for 5 days. To grow spherules, arthroconidia were washed 2 times in modified Converse media [12]. The spores were inoculated at 4×10^6^ arthroconidia/ml into a 250 ml baffled Erlenmeyer flask containing 50 ml of modified Converse media. Eight identical flasks were set up and grown on a shaker at 160 rpm, in 14% CO_2_ at 42°C. Four flasks were harvested 2 days after inoculation and the remaining four flasks after 8 days. The spherules did not rupture and release endospores within that time in this culture system.

### Inhibition of growth with nitisinone

Nitisinone, 2-(2-nitro-4-trifluoromethylbenzoyl)-cyclohexane-1, 3 dione, a potent specific inhibitor of 4-HPPD was purchased from Swedish Orphan Biovitrum, Sweden. A stock solution of 30 mg/ml was made in 0.2 M NaOH. Nitisinone was added at several concentrations to glucose yeast extract media (GYE) or modified converse media in the presence of 2×10^6^ spores/ml in a 15 ml round-bottom tissue culture tubes (BD Falcon). The culture was grown as described above for mycelial and spherule growth. The control tubes contained equal amounts of 0.2 M NaOH without Nitisinone. For microscopy, 1% formaldehyde was added to the culture overnight and the tubes were centrifuged 10,000 rpm for 10 min. The pellet was re-suspended in Lactophenol Aniline blue stain (Remel) and examined microscopically.

### RNA isolation

*C. immitis* mycelia were harvested by straining the media from four cultures through a 40 μM nylon cell strainer (BD Falcon). The mycelia were picked up from the cell strainer using a sterile disposable loop (BD Falcon) and dropped in a 2 ml ZR BashingBead lysis tube with 0.5 mm beads (Zymoresearch) and 0.5 ml Qiazol reagent (Qiagen). The tubes were arranged in a pre-cooled Tissuelyzer II adapter (Qiagen) and mycelia was disrupted by shaking at 50 Hz for 25 min. Spherules in Converse media were harvested from four 2 day cultures and four 8 day cultures. The cell concentration was determined by counting the spherules in Lactophenol Aniline blue stain. The media was centrifuged at 10,000 rpm for 10 min at 4°C. Qiazol (Qiagen) was added to the cell pellet at 4×10^6^ spherules/ml and 0.5 ml of the mixture added to a 2 ml ZR BashingBead lysis tube with 0.5 mm beads (Zymoresearch). Total RNA was purified from mycelia and spherule samples (4 replicates/condition) using the RNeasy Microarray tissue mini-kit (Qiagen) in a Qiacube machine (Qiagen). If necessary RNA was concentrated or re-purified using RNeasy Minelute Cleanup kit (Qiagen) according to the manufacturer’s protocol. Total RNA concentrations were initially determined using a NanoDrop spectrophotometer (NanoDrop) and RNA quality was assessed using a 2100 Bioanalyzer (Agilent). All samples had a RNA integrity number greater than 7.

### Microarray design and hybridization

Known and predicted ORFs from the *C. immitis* genome (RS strain) were previously identified using sequence data available at the Broad Institute [14]. This information was supplied to Roche Nimblegen in order to manufacture a custom oligonucleotide array consisting of 68,927 probes (Nimblegen custom array OID30589). Probes were 60 nucleotides in length and the expression of the majority of genes was assayed using 7 different probes printed in duplicate. The expression of small genes was assayed with fewer probes. Twelve custom microarrays fit on a single slide such that all the samples in this study (4 × mycelia, 4 × day 2 spherule, and 4 × day 8 spherule) could be assayed for gene expression in a single experiment to eliminate technical batch effects. Ten μg of total RNA at a concentration greater than 1 μg/ml from each sample was used for microarray hybridization. Total RNA was converted to cDNA, labeled with dye, and hybridized to the microarray by the VA San Diego Gene Chip Microarray Core according to the Nimblegen protocol. All *C. immitis* genes are referred to by their locus tag and further information about these genes can be found at the Coccidioides group database at the Broad Institute http://www.broadinstitute.org/annotation/genome/coccidioides_group/MultiHome.html. FungiDB (http://fungidb.org/fungidb/) was also used for annotation because it has more informative gene names for many genes.

### Microarray data analysis

Quality control analysis and normalization of microarray gene expression data were performed as previously described [15]. Briefly, several quality control assessments (e.g., boxplots and volcano plots) were applied to assess microarray data quality. Unsupervised clustering was also performed using the web-based tool ANAIS [16] to determine if samples clustered as expected based on the expression of genes in each sample. All arrays passed quality control filters and no outliers were found. Differentially expressed probes were identified between mycelium, day 2 spherule and day 8 spherule conditions using a one-way ANOVA and the Tukey *post hoc* test implemented in GeneSpring GX version 11.5 (Agilent Technologies Inc.). The false discovery rate (FDR) associated with multiple tests was corrected for using the Benjamini-Hochberg method [17]. In a conservative approach, a gene was only identified as differentially expressed if all probes for that gene had a fold change greater than 2 or less than −2 and an ANOVA *p*-value (Tukey and FDR corrected) less than 0.05. Fold changes were calculated for each gene that passed this filter by averaging across the seven probes. The overlap in up and downregulated genes between day 2 and day 8 spherules compared to mycelia was visualized in Venn diagrams using BxToolBox (http://bioinforx.com/). The ANOVA analysis was also used to identify genes that were differentially expressed between day 2 and day 8 spherules where positive fold changes are indicative of greater expression at day 8 compared to day 2 and negative fold changes suggest decreased expression. Gene expression data are available at the Gene Expression Omnibus (http://www.ncbi.nlm.nih.gov/geo/) under accession number GSE44225.

### PFAM and GO analysis

PFAM enrichment was determined using a tool at the Broad Institute http://www.broadinstitute.org/annotation/genome/coccidioides_group/BatchSelect.html?target=GeneEnrichment.html. This tool looks for over-representation of PFAMs in up- or downregulated genes using a hypergeometric test, and only PFAMs with an FDR-corrected p-value <0.05 were considered significant.

GO terms were assigned to *C. immitis* genes by reciprocal homology searches at the protein level against the *Saccharomyces cerevisiae* proteome using BLAST (Additional file 1: Table S1). UniProt IDs were obtained using the *C. posadasii* homologs of *C. immitis* genes because many more *C. posadasii* genes have UniProt IDs. The Biological Networks Gene Ontology (BiNGO) plugin (version 2.441) [18] for Cytoscape (version 2.8.3) was used to identify those GO terms related to biological processes that were over-represented for differentially expressed genes identified between each of the three comparison groups (mycelia vs. day 2 spherule, mycelia vs. day 8 spherule, day 8 vs. day 2 spherule). BiNGO preserves the hierarchical relationship between GO terms. Significance was assessed with a hypergeometric test and only GO terms with an FDR-corrected p-value <0.05 were considered significant.

### Gene annotation

*C. immitis* protein kinases were identified and classified by orthology with the curated *Trichophyton rubrum* kinome [19]. Non-orthologous kinases were identified and classified by searching the proteome with a protein kinase HMM built from an alignment of *Dictyostelium* kinases [20] followed by a BLAST against the curated kinase database (http://kinase.com/) [21]. Kinase abbreviations are provided in Additional file 2: Table S3. Signal peptides in the proteins coded for in the *C. immitis* genome were identified using artificial neural networks implemented in SignalP version 4.0 [22].

### RT-qPCR confirmation of gene expression

Microarray gene expression was confirmed by RT-qPCR for 24 genes. Three highly expressed genes with low standard deviation across the 12 samples were selected as normalizers (CIMG_01599, CIMG_10083 and CIMG_12902). SYBR® Green primers were designed using Primer Express version 3.0 (Applied Biosystems Inc.) and obtained from Integrated DNA Technologies, Inc. (Coralville, IA). Reverse primers were designed to span a splice site in the same region of the gene probed by the microarray. Duplicate RT-qPCR reactions were performed for each sample using 50 ng of reverse transcribed RNA per reaction. Fold changes were calculated as described previously using the 2^-∆∆CT^ method [23] implemented in the DataAssist software version 3.0 (ABI), and significance was determined using one-way ANOVA in the R statistical package (version 2.13.2).

## Results and discussion

### Genes differentially expressed in mycelia and spherules

**Figure 1:**
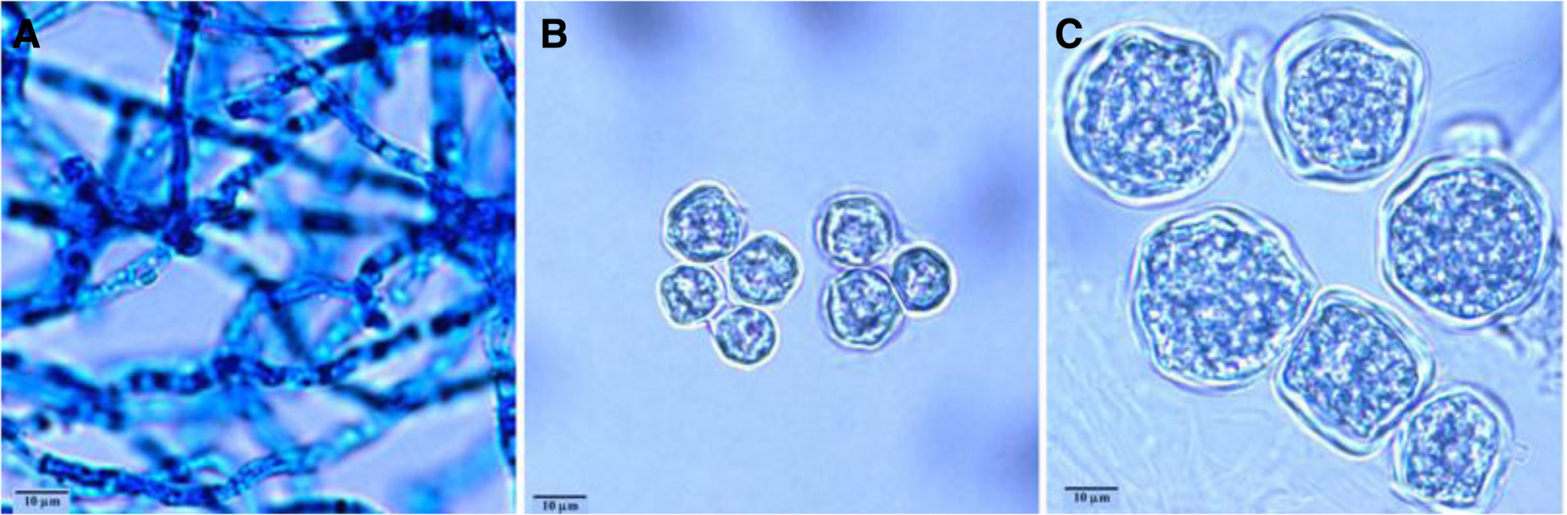
**Photomicrographs of C. immitis strain RS mycelium and spherules after 2 and 8 days of culture.** Notice the large increase in size as the spherules mature.

Gene expression was assessed in a total of 12 samples derived from 4 replicate samples isolated from the following three growth phases: mycelia, day 2 spherules, and day 8 spherules. A photograph of mycelia and day 2 and day 8 spherules grown in Converse medium is shown in Figure 1. The image shows the difference in shape and size between spherules and mycelia and the increase in spherule size between 2 and 8 days of culture. A custom oligonucleotide microarray (Nimblegen), which contained probes for all predicted ORFs of the RS strain of *C. immitis* was used to assess gene expression. 91% of the predicted ORFs were expressed in either mycelia or spherules, suggesting that the annotation and the detection of hybridization were robust. Unsupervised clustering using the expression of all genes on the microarray revealed that mycelia samples clustered distinctly from spherule samples. Furthermore, spherule samples formed two sub-clusters based on the number of days in culture. A dendrogram showing that the four replicate samples cluster together is shown in Additional file 3: Figure S1. Fungal morphologic stage was the dominant determinant of the pattern of gene expression.

**Figure 2:**
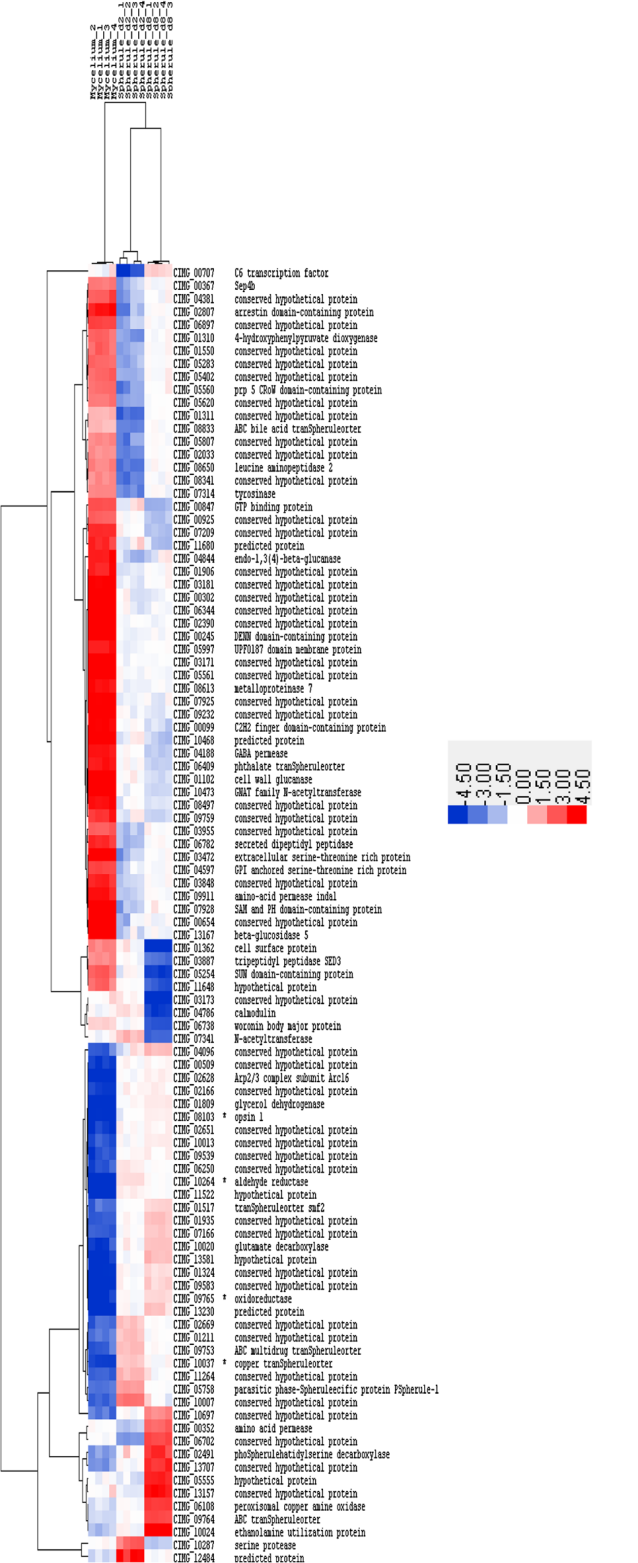
**A heatmap depicting expression levels of genes in mycelia, day 2 spherules and day 8 spherules for the top 100 genes that were differentially expressed between mycelia, day 2 spherules and day 8 spherules.** Fold changes were calculated for day 2 spherules vs mycelia and day 8 spherules vs mycelia. For each gene, the absolute peak log 2 fold change (FC) was identified across the three conditions and the raw expression values for the top 100 were log transformed and median-centered and included in the heatmap. Hierarchical clustering of genes and array samples based on their expression profiles is reflected in the dendrograms to the left and the top of the heatmap respectively and was performed by calculating distances using the Pearson correlation metric and then clustering distances using the average linkage method. The expression of genes marked with an asterisk (*) was confirmed by RT-qPCR. The scale is shown: red shading indicates greater expression blue shading represents lesser expression.

**Figure 3:**
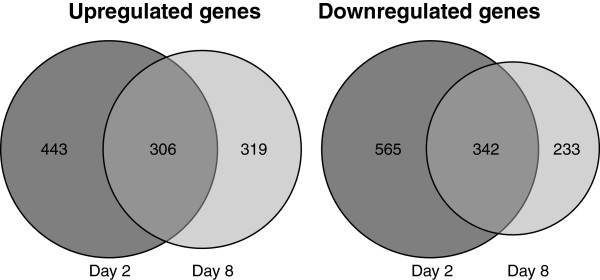
**Venn diagrams showing the number of genes that are differentially expressed in day 2 spherules and day 8 spherules compared to mycelia.** The number of up- or downregulated genes in shown. The procedures for determining up- or downregulation are in the methods section.

Genes that were significantly differentially expressed (p < 0.05) between the three conditions (mycelia, spherules on day 2 and 8) were identified in a supervised approach using a one-way ANOVA with appropriate corrections for multiple testing (see Methods). All the up- and downregulated genes differentially expressed between each of the three conditions are detailed in Additional file 4: Table S2. A heatmap depicting expression levels in each sample for the top 100 differentially expressed genes is presented in Figure 2. The heatmap indicates there was limited variation in gene expression across the four replicates within each of the three conditions, suggesting that the data was highly reproducible. Multiple patterns of gene expression are evident comparing the three different conditions we studied. One cluster of genes was expressed to a lesser extent in the mycelia condition and a greater extent in both spherule conditions and another cluster of genes were expressed at a higher level in mycelia than in spherules. The expression of four genes (CIMG_08103, CIMG_09765, CIMG_10037, CIMG_10264) exhibiting the upregulated pattern was confirmed by RT-qPCR (see Figure 3 below).

**Figure 4:**
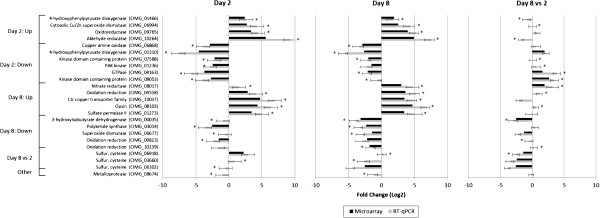
**Confirmation of gene expression differences by RT-qPCR between day 2 spherules vs mycelia, day 8 spherules vs mycelia and day 8 vs day 2 spherules.** The figure shows a comparison between the fold change for each gene for RT-qPCR data (grey bars) and microarray data (black bars) between the different conditions. RT-qPCR gene expression data (2^-∆∆CT^) was averaged within conditions and then used to calculate log_2_ fold change values between the different conditions for direct comparison to microarray data. A positive fold change indicates the gene was expressed to a greater extent within a condition. An asterisk (*) indicates that the gene was significantly differentially expressed (*p* <0.05, *t*-test) and the error bars on the RT-qPCR data represent the standard deviation between the biological replicates of mycelia, spherules at day 2 and spherules at day 8.

There were a total of 2208 genes (22% of the genome) that were differentially expressed between spherules at one or both the time points we studied and mycelia. Figure 4 shows Venn diagrams depicting up- and downregulated genes in day 2 and day 8 spherules compared to mycelia. About a third of the differentially expressed genes were up- or downregulated in both day 2 and day 8 spherules compared to mycelia. However, similar numbers of genes were exclusively upregulated in either day 2 (N = 443) or day 8 (N = 319) spherules, or exclusively downregulated at either day 2 (N = 565) or day 8 (N = 233) spherules. The difference in gene expression between day 2 and day 8 spherules was apparent when we compared day 2 and 8 spherules directly to each other; 1,197 differentially expressed genes (12% of the total genome) were identified (Additional file 4: Table S2). Therefore, although gene expression by environmental form of the fungus and the parasitic form were quite distinct as might be expected, gene expression by young and mature spherules was also quite different from each other. Not only were there differences in which genes were expressed at each stage, but also the degree of modulation was large. For example, the maximum difference in expression of a gene (CIMG_10264) between day 2 spherules and mycelia was 48.6 fold and the median modulation between mycelia and day 2 spherules was 3.26.

A recent paper by Whiston et al. assessed transcription in *C. immitis* and *C. posadasii* mycelia and day 4 spherules by RNA-seq [13]. We have compared our results to theirs. The two studies used different methods for assessing changes in gene expression. We used microarray technology to estimate transcript abundance while Whiston et al. used RNA-seq to estimate transcript abundance [13]. The literature suggests that these methods should yield comparable results [24]. Despite this difference in methodology, we confirmed the upregulation of 25% of the genes that Whiston found to be upregulated in spherules. Conversely, 43% of genes that we have found to be upregulated in day 2 and day 8 spherules were also upregulated in day 4 spherules in the Whiston study (Additional file 5: Figure S2). Despite the differences in the two studies many of our conclusions are similar (see below).

We know from previous experiments that some genes are overexpressed in spherules compared to mycelia. Some of these genes, such as the spherule outer wall glycoprotein (CIMG_04613) [25] and the parasitic-phase specific protein *PSP-1* (CIMG_05758) [26] were up regulated more than four fold in spherules in this experiment (Additional file 4: Table S2). Other genes, such as the metalloproteinase *Mep1* (CIMG_06703), which has been found to be expressed at high levels in endosporulating spherules in *C. posadasii* was not found to be over-expressed in this experiment [27]. We also examined the expression level of the *Mep1* gene by RT-qPCR and found that its expression was slightly downregulated in spherules compared to mycelia, rather than upregulated as previously reported (see below). Whiston et al. also examined the expression of this gene and found that it was upregulated in *C. posadasii* spherules but not *C. immitis* spherules [13].

### Confirmation of differential expression by RT-qPCR

Twenty-four differentially expressed genes as detected by microarray analysis were selected for confirmation by RT-qPCR (Figure 3). Genes were selected for RT-qPCR confirmation of gene expression based on the magnitude of fold change (up- or downregulation) between mycelia and day 2 spherules, mycelia and day 8 spherules, and day 2 and day 8 spherules, and their identification in the PFAM or GO analysis. The significant differential expression (p < 0.05, *t*-test) of each of these 24 genes was confirmed for at least one of the three comparison groups. In the majority of cases, RT-qPCR analysis detected larger differences in expression than microarray analysis. This is probably because RT-qPCR has a greater dynamic range than microarray does [28].

### PFAM analysis

#### Day 2 and day 8 spherules vs mycelia

Functional enrichment analysis of PFAM families is shown in Table 1. Genes in the thioesterase superfamily are upregulated in day 2 spherules compared to mycelia. This family of proteins hydrolyzes long chain fatty acyl-CoA thioesters and is also involved in hydrolysis of fatty acids from S-acylated cysteine residues in proteins, with a strong preference for palmitoylated G-alpha proteins over other acyl substrates [29]. Upregulation of genes involving lipid metabolism is reasonable since spherules contain a much higher percentage of lipids than mycelia [30].

**Table 1:** PFAM functional enrichment

**PFAM Term**	**# Specified Genes**	**Whole Genome**	**Specified Gene %**	**Whole Genome %**	**P Value**	**Corrected P Value**
**Day 2 Spherules Upregulated**						
Thioesterase superfamily (4HBT)	5	6	0.99	0.06	0.0	0.0010
Short chain dehydrogenase	11	57	2.19	0.58	0.0	0.04
Aldol-keto_reductase	6	13	1.19	0.13	0.0	0.0080
**Day 8 Spherules Upregulated**						
MFS_1	14	140	3.85	1.43	0.0	0.131
Aldol-keto_reductase	5	13	1.37	0.13	0.0	0.018
**Day 8 vs 2 Spherules Upregulated**						
C2	6	11	0.96	0.11	0.0	0.0090
PHD	8	16	1.29	0.16	0.0	0.0010
SH3_1	8	24	1.29	0.25	0.0	0.027
Pkinase	21	92	3.38	0.94	0.0	0.0
**Day 2 Spherules Downregulated**						
PH	8	16	0.93	0.16	0.0	0.011
SH3_1	14	24	1.63	0.25	0.0	0.0
SH3_2	11	18	1.28	0.18	0.0	0.0
Pkinase	23	92	2.68	0.94	0.0	0.0010
zf-C2H2	19	53	2.21	0.54	0.0	0.0
**Day 8 Spherules Downregulated**						
Kinesin	6	10	1.14	0.1	0.0	0.0020
**Day 8 vs 2 Spherules Downregulated**						
None						

The short chain dehydrogenases family was also upregulated in day 2 spherules (maximum upregulation 10.27 fold, CIMG_09765). This family of enzymes catalyzes oxidation/reduction reactions of alcohols and cyclic compounds. Up regulation of this family of enzymes seems plausible given the shift in growth conditions from air which contains less than 0.05% CO_2_ (mycelial growth) to 14% CO_2_ (spherule growth) which mimics the shift in oxidation/reduction potential that occurs when the organism grows in the mammalian host. The aldol-keto reductase family was also significantly enriched in both day 2 and day 8 spherules. These genes were also found to be upregulated in spherules by Whiston et al. [13]. This protein family plays a role in reducing oxidative stress [31] and may be required for resistance to the oxidative burst in mammals or to mitochondrial generated reactive oxygen species. *C. immitis* spherules are more resistant to oxidative killing *in vitro* than *Aspergillus fumigatus* spores [32].

The major facilitator super family (MFS-1) that was enriched in day 8 spherules is an important transporter of small molecules and includes a number of transporters for uptake as well as efflux [33]. Two highly upregulated genes are sugar transporters (CIMG_03001 and CIMG_08310). A nicotinic acid transporter (CIMG_06071) and a sialate transporter (CIMG_03956) were also upregulated. The nicotinic acid transporter is presumably involved in NAD metabolism [34]; we have been unable to find a role for the sialate transporter in fungi in the literature.

The pleckstrin domain occurs in a wide range of proteins involved in intracellular signaling or as constituents of the cytoskeleton. Pleckstrin domain transcripts were downregulated in day 2 spherules; in fact, one pleckstrin domain gene is the most downregulated of all the day 2 genes (CIMG_07982, -53.53 fold). The downregulated pleckstrin domain containing genes may be required for polar mycelial growth but not isotropic spherule growth. One downregulated gene in this family is the anucleate primary sterigmata protein A (CIMG_06141, -4.93), which is critical for movement of nuclei into spores on the sterigmata of *A. nidulans*[35]. This gene may well be required for arthroconidia formation in *C. immitis* mycelia but not endospore formation in spherules.

A significant proportion of proteins containing SH3 domains were downregulated in day 2 spherules. SH3 protein families include some protein kinases, phosphoinositol 3 kinases, Ras GTPase activating proteins, and the guanine nucleotide exchange factors *cdc24* and *cdc25*[36]. Two of these genes, CIMG_04361 and CIMG_04531, were downregulated in day 2 spherules. CIMG_04531 is annotated as a polarized growth protein, and is highly homologous to cytoskeleton assembly proteins in many fungi. CIMG_02193 is cytoskeletal protein *SLA1* and it is downregulated (−4.61 fold change) in day 2 spherules. Perhaps these proteins predispose to polar mycelial growth rather than isotropic spherule growth.

On the whole, the protein kinase family is downregulated in day 2 spherules. (This gene family was also detected by GO enrichment analysis in day 2 spherules but the p-value did not achieve significance with the BH correction. The two analyses identified almost identical sets of genes.) Examining the up- and downregulated genes, we found that 23 genes were downregulated (−7.84 to −2.71 fold) and only two were upregulated (4.55 to 2.48 fold) (Table 2). Whiston et al. also found that 10 of these protein kinase genes were downregulated in spherules [13]. Four of the most downregulated genes were homologs of *S. cerevisiae* genes involved in sex or meiosis (indicated by an asterisk in Table 2). *C. immitis* has all the genes required for a sexual cycle [37] and has been shown to recombine in nature [38], but the sexual cycle has never been observed. Six of the downregulated protein kinase genes were homologs of *S. cerevisiae* genes involved in mitosis (indicated by a double asterisk in Table 2). Presumably some of these genes may interfere with arthroconidia conversion to spherules. The idea that there is more DNA replication in mycelia than in spherules has been previously proposed [5]. Of the two upregulated kinases, only CIMG_05990 (GCN2) has an ortholog in budding yeast. This kinase phosphorylates eIF2 in response to amino acid starvation [39]. Increased expression of GCN2 coupled with decreased expression of CIMG_08909, a sky1p ortholog involved in mRNA splicing [40], is consistent with the hypothesis that the rate of protein production in day 2 spherules is lower than in mycelia Additional file 2: Table S3 lists the functional classification of all of the 184 *C. immitis* protein kinases and their *S. cerevisiae* homologs. 126 of these are eukaryotic protein kinases (ePKs) and 58 are atypical protein kinases (aPK). Of the ePK there are 47 novel kinases: 17 SRPKLs (serine/arginine rich protein kinase-like), 6 PezKs (pezizomycotina kinases) and 24 unclassified kinases designated as ‘Other’. We believe these 47 kinases to be novel because we did not observe orthologs in the species used for comparison, and they do not match families in kinase.com. There are 38 aPKs from well-known families, and 20 FunK1s (fungal kinase 1s) from a family recently described in *Coprinopsis cinerea*[41] and *Paracoccidioides*[42]. Examining the classification of the differentially expressed protein kinases in day 2 spherules we found that 50% of *STE11* kinases, 40% of the *STE20* kinases and none of the *STE7* kinases were downregulated compared to mycelia. 40% of the CAMK/CAMKL kinases are downregulated. Although the numbers are small, most of the protein kinases in the other/WEE, other/RAN and other/NAK classifications were downregulated.

**Table 2:** Modulated protein kinases in day 2 and day 8 spherules

**Gene ID**	**FC**^**a**^	**FC**^**b**^	***C. immitis*annotation**	**Classification gene**	**S. cerevisiae**
CIMG_05093	−7.84	2.78	Serine/threonine-protein kinase; meiosis induction protein kinase	CMGC/RCK/MAK	*IME2* *
CIMG_09053	−6.68	6.18	Kinase domain containing protein	CAMK/NNK1	*NNK1*
CIMG_07296	−5.60	5.26	Protein kinase domain containing protein	CAMK/CAMKL/MARK	*YPL150W*
CIMG_01236	−5.46	--	PAK kinase	STE/STE20/PAKA	*STE20* *
CIMG_00940	−5.28	--	Protein kinase	Other/WEE/SWE1	*SWE1* **
CIMG_07521	−4.67	2.94	Protein kinase domain containing protein; serine/threonine protein kinase 24	STE/STE20/YSK	*SPS1* *
CIMG_04027	−4.65	3.81	serine/threonine protein kinase ssp1	Other/CAMKK	None
CIMG_03267	−4.55	--	serine/threonine protein kinase	CAMK/CAMKL/Kin4	*KIN4* **
CIMG_07588	−4.52	--	Kinase domain containing protein; checkpoint kinase	Other/TTK	*MPS1* **
CIMG_01204	−4.34	4.02	protein kinase	AGC/YANK	None
CIMG_08909	−4.14	3.06	Protein kinase, sky 1	CMGC/SRPK	*SKY1*
CIMG_03947	−4.04	3.64	serine/threonine protein kinase	CAMK/CAMKL/PASK	*PSK1*
CIMG_03602	−3.98	3.70	Ran1-like protein kinase	Other/RAN/VHS1	*VHS1* **
CIMG_04103	−3.97	--	cytokenesis protein sepH	STE/STE11/CDC15	*CDC15* **
CIMG_08220	−3.96	6.13	serine/threonine protein kinase ATG1	Other/ULK/ULK	*ATG1*
CIMG_06932	−3.81	2.58	MAP kinase kinase kinase SskB	STE/STE11/MEKK4	*SSK2*
CIMG_13010	−3.74	3.93	serine/threonine protein kinase	Other/RAN/KSP1	*KSP1* *
CIMG_09191	−3.52	2.50	Protein kinase	Other/HAL/HRK1	*HRK1*
CIMG_09469	−3.36	--	Kinase domain containing protein	Other/PEK	None
CIMG_03857	−3.08	--	Kinase domain containing protein	Other/NAK/BIKE	*PAK1*
CIMG_02369	−3.05	--	Response regulator receiver RIM15p	AGC/NDR/RIM15	*RIM15* **
CIMG_05623	−2.74	--	Serine threonine protein kinase	CAMK/CAMKL/AMPK	*SNF1*
CIMG_00136	−2.71	--	Kinase domain containing protein	CMGC/DYRK/DYRK2	*YAK1*
CIMG_02925	−4.55	--	Protein kinase domain containing protein	CMGC	None
CIMG_05694	3.01	−3.83	Protein kinase domain containing protein	CMGC/SRPKL1	None
CIMG_05990	2.48	--	RWD domain protein	Other/PEK/GCN2	*GCN2*

* Indicates a gene involved in the sexual cycle in Saccharomyces; ** Indicates a gene involved in mitosis in Saccharomyces; a) Indicates a comparison between day 2 spherules and mycelia; b) indicates a comparison between day 8 and day 2 spherules; --, indicates that the gene is not modulated.

C2H2 zinc finger domain containing proteins were downregulated in day 2 spherules. Most of the proteins containing this domain are transcription factors and the zinc finger is involved in DNA binding [43,44]. Some of the genes that were downregulated include the transcription factors CIMG_04642 (−9.24, *FlbC*), CIMG_03725 (−5.06, zinc finger transcription factor *PacC*) and CIMG_06050 (−3.06, transcription factor *steA*). In fact, *steA* is a negative regulator of transcription in Aspergillus, so downregulation of this gene probably results in upregulated transcription of some genes [45]. Ste12 is the Saccharomyces homolog of this gene [46]. Ste12 is involved in the mating response and is involved in the up- and downregulation of many genes. Eight of these 19 C2H2 zinc finger genes were also found to be downregulated in spherules by Whiston et al. [13].

#### Day 8 spherule/day 2 spherule comparison

Several of the gene families that were downregulated in day 2 spherules were upregulated in the day 8 spherules (Table 1). Examples are the Ras GTPase activating proteins, the guanine nucleotide exchange factors *cdc24* and *cdc25*[36]. 13 of 19 of the kinases downregulated in the day 2 spherules had returned to mycelial levels in day 8 spherules (Table 2). Two genes in this family were downregulated in both day 2 and day 8 spherules: CIMG_00940 (−5.28 fold in day 2 spherules and −10.04 in day 8 spherules both compared to mycelia) and CIMG_04103 (−3.97 fold in day 2 spherules and −6.75 in day 8 spherules both compared to mycelia). CIMG_00940 was also found to be downregulated in spherules by Whiston et al. [13]. CIMG_00940 is a Swe1 kinase and CIMG_04103 is a STE/STE11/CDC15 kinase. Both of these genes are involved in regulation of mitosis [47-49]. One function of Wee kinases in *S. cerevisiae* is to prevent small cells from entering mitosis [50]; endospores are very small so downregulation of this gene may be important for endospore division. A function of the CDC15 kinases is to bind the spindle pole body and facilitate exit from mitosis [49]. There is no obvious reason why this kinase should be downregulated in the internally dividing spherule.

### Gene ontology analysis

#### Mycelia/day 8 spherules comparison

The gene ontology (GO) terms significantly over-represented in lists of differentially expressed genes identified between mycelia and either day 2 or day 8 spherules, as well as between day 2 and day 8 spherules, were identified using BiNGO [18]. There were no GO terms that survived FDR correction between mycelia and day 2 spherules but a large number of significant terms were identified between mycelia and day 8 spherules (Additional file 6: Figure S3). The most significant enriched GO term was “small molecule metabolic process” (corrected p = 0.004). Thirty-one members of this heterogeneous set of genes were upregulated and 75 were downregulated. Twelve of the downregulated genes coded for nucleotide synthesis or DNA replication. For example, a homeobox domain-containing protein was downregulated −8.68 fold (CIMG_09071); thymidylate synthase was down −3.57 fold (CIMG_08646); cell division control protein *Cdc6* was down −3.05 fold (CIMG_07523) and DNA topoisomerase 2 was down −3.09 fold (CIMG_02836). This suggests that the rate of DNA synthesis is slower in the day 8 spherules than in mycelia. 10 genes coding for amino acid synthesis were downregulated as well. This suggests that not only is DNA synthesis relatively slow compared to mycelia but protein synthesis is too. Other genes involved in vitamin synthesis and energy generation were also downregulated. This is consistent with the notion that day 8 spherules have produced their endospores. Rupturing and releasing endospores should not be a metabolically expensive process. The observation that MFS-1 sugar transporters are upregulated suggests that that the low metabolic needs may not be universal.

The most strongly upregulated genes in day 8 spherules with the GO term “small metabolic process” included glutamate decarboxylase (21.47), three ABC transporters and parasitic phase specific protein-1 (6.66) previously described by Delgado [26]. The *PSP-1* gene is also upregulated in day 2 spherules and in day 4 spherules as reported by Whiston et al. [13]. *PSP-1* contains a RTA-1 domain, which is involved in resistance to 7-aminocholesterol [51]. This family of proteins has multiple membrane spanning domains and is thought to be involved in binding 7-aminocholesterol and related substances and preventing toxicity. They are not thought to be efflux pumps [51].

A group of genes assigned the GO term “carbohydrate metabolic processes” was also enriched in the day 8 spherules dataset. 15 genes were upregulated and 17 genes were downregulated. The upregulated genes included polysaccharide deacetylase (CIMG_02628, 34.82) and 1,4 (α)-amylase (CIMG_03529, 2.70). The most striking downregulated gene in this group is calmodulin (CIMG_04786, -10.38). Two other genes coding for calmodulin (CIMG_02413 and CIMG_08162) are not differentially expressed in day 8 spherules. We looked for differential expression of six calmodulin-dependent kinases and found that they were not up- or downregulated. Calmodulin-dependent signaling enzymes are required for growth of *Cryptococcus neoformans* at 37°C [52] and for conversion of *Sporothrix shenkii* from mold to yeast [53].

Another enriched GO term in day 8 spherules was “oxidation-reduction processes” (corrected p-value = 0.012). In this group of genes, twenty-seven genes were upregulated in spherules and 27 were downregulated in spherules compared to mycelia. The five most upregulated genes were aldol-keto reductases (maximum 30.2 fold), alcohol dehydrogenases (maximum 11.65 fold) and nitrate reductase (8.06). The aldol-keto reductases were also identified in the PFAM functional enrichment in day 2 and day 8 spherules (Table 1). All these responses make sense in terms of the difference in growth conditions of mycelia (grown in air containing less than 0.05% CO_2_) and spherules (grown in 14% CO_2_ in air). These responses also seem reasonable in terms of the spherule living in the high CO_2_ environment of the mammalian host compared to the mycelia living in the soil. Other upregulated genes include *Y20* (CIMG_04756), which was upregulated 11.18 fold in day 2 spherules and 6.81 fold in day 8 spherules. This gene was also upregulated in spherules in the Whiston study [13]. This gene codes for a flavodoxin that plays a role in the cellular responses to oxidative stress [54]. A homolog of this gene is highly upregulated in the yeast phase of *P. brasiliensis*[54]. Presumably this protein is protecting the fungus against oxidative attack by the mammalian host. Additional genes coding for response to oxidative stress that were upregulated in day 8 spherules include a Cu/Zn super-oxide dismutase (CIMG_06994, 5.88) and a catalase. CIMG_06994 was up regulated 6.51 fold in day 2 spherules too. This gene is highly homologous to the extracellular *SOD3* (e = 4 × 10^-50^) recently identified as a secreted protein in the *Histoplasma* yeast phase [55]. Gene deletion experiments have shown that this gene is important for defense against oxidative stress [56]. *SOD3* is a secreted protein in *H. capsulatum*; CIMG_06944 has a predicted signal sequence suggesting that it is a secreted protein too. Extracellular SOD may be more protective against mammalian oxidative stress as suggested by Youseff [56]. Presumably the *C. immitis* homolog of *SOD3* is up regulated to protect the spherule against oxidative stress in the host. *C. immitis* also contains genes highly homologous to *A. fumigatus SOD2* and *SOD4* but neither of those is up- or downregulated.

Several NAD or NADPH dependent oxireductases were downregulated 3–8 fold in day 8 spherules. An NADPH oxidase was downregulated 3.48 fold. This enzyme makes reactive oxygen intermediates in fungi just as it does in mammalian phagocytes [57]. Reactive oxygen intermediates are important for apical growth and formation of spores in filamentous fungi [57,58]. It is possible that the NADP oxidase may interfere with isotropic spherule growth and differentiation.

#### Day 8/day 2 spherule comparison

Several GO terms were significantly over-represented (FDR corrected p-value <0.05) in the list of genes differentially expressed between day 8 and day 2 spherules. The most significant enriched GO term was “sulfur compound metabolic process” (corrected p-value = 0.046). Sixteen genes were downregulated in this data set and one was upregulated. We see downregulation of 5′-methylthioadenosine phosphorylase (CIMG_01361, -7.45 fold), phosphoadenosine phosphosulfate reductase (CIMG_00456, -4.65 fold), two genes coding for adenylyl-sulfate kinase (CIMG_00454, -4.22 fold and CIMG_06918, -2.65 fold) and sulfite reductase (CIMG_00269, -2.94 fold) in day 8 spherules. Two of these genes were upregulated in day 2 spherules compared to mycelia. All these genes are involved in accumulating sulfide. This suggests that *C. immitis* spherules have no difficulty accumulating enough sulfur for their needs as they mature.

### Upregulated or downregulated genes in day 2, day 4 and day 8 spherules

We identified 153 genes that were upregulated more than two fold in day 2 spherules, day 8 spherules and day 4 spherules in the Whiston study [13]. 140 genes were downregulated more than two fold in all three spherule samples. 57% of the upregulated genes and 42% of the downregulated genes had no function annotation (Additional file 7: Table S4). Many of these unannotated genes were highly differentially expressed suggesting that they may be important for spherule development. One upregulated gene that has not been discussed is opsin-1 (CIMG_08103, maximal upregulation 25.72). This gene is closely related to the bacterial rhodopsin gene coding for G protein coupled receptors; its function in fungi has not been determined [59]. Another gene that was upregulated at all three spherule time points was the sulfite transporter Ssu1 (CIMG_05899, maximum upregulation 6.37). Downregulated genes of interest include several glucosidases, glucanases and a chitosanase. Two septin genes are downregulated in spherules. Septin genes are important regulators of the cytoskeleton and play a role in determining cell shape [60]. Why these genes are downregulated is unclear since the spherule is undergoing extensive cellular remodeling. Perhaps septins are required for polar growth and other regulators are needed for isotropic spherule growth. Further analysis of the relatively small group of genes that are consistently up- or downregulated throughout spherule development may be useful for understanding the pathogenic phase of this organism.

### Comparison of results to those obtained in other pathogenic fungi

The dimorphic pathogenic fungi are phylogenetically closely related [61] so it is reasonable to ask if genes important for conidium to yeast transformation in those pathogens are also important for arthroconidia to spherule transformation in *Coccidioides*. One *H. capsulatum* gene that is required for mycelium to yeast transformation is the alpha amylase (*AMY1*) gene. This gene affects the amount of α (1,3) glucan in the yeast cell wall, and that carbohydrate appears to be important for the ability of the yeast to survive in macrophages and in mice [62]. This gene has a nearly identical homolog in *C. immitis*, CIMG_03142, that was upregulated 3.6 fold in day 2 spherules and 3.39 fold in day 8 spherules. Whiston et al. also found it to be upregulated in spherules [13]. Another *H. capsulatum* gene that is required for yeast formation is α glucan synthase (*AGS1*) gene [62]. This enzyme catalyzes the production of α (1,3) glucan in the cell wall that obscures the β (1,3) glucan and prevents activation of innate immunity via the dectin-1 receptor [62]. *C. immitis* has an *AGS1* gene (CIMG_13256) that was upregulated in the day 8 spherule (2.48 fold) but not day 2 spherules. Whiston et al. found this gene to be upregulated 1.93 fold in spherules compared to mycelia [13]. There is no literature describing the relative amounts of α (1,3) glucan and β (1,3) glucan in *C. immitis* mycelia or spherules. We know, however, that there is enough exposed β (1,3) glucan in *Coccidioides* spherules to stimulate macrophages to produce cytokines via dectin-1 [63].

Two genes coding for transcription factors, *Ryp2* and *Ryp3*, have been found to be essential for conversion from filaments to yeast in *H. capsulatum*[64]. These genes are overexpressed in the yeast phase of *H. capsulatum*[64]. *C. immitis* has nearly identical homologs of these genes but they were not overexpressed in either day 2 or day 8 spherules, suggesting that they may not be required for the transformation from mycelium to spherule.

Gene disruption experiments in *B. dermatitidis* have shown that a histidine kinase, *DRK1*, is required for the transformation from filaments to yeast [65]. It is not clear from the literature whether or not this gene is overexpressed in the *B. dermatitidis* yeast phase. *C. immitis* has a very closely related homolog of this gene (CIMG_04512) but it was not up or down regulated in day 2 or day 8 spherules. In another dimorphic pathogenic fungus, *S. schenckii*, the calcium/calmodulin kinase I gene (*SSMK1*) was found to be required for formation of yeast [53]. There are two genes in *C. immitis* that are highly homologous to the *S. schenckii SSMK1* gene; neither one of these was up- or downregulated in day 2 or day 8 spherules.

A number of studies have been done studying the transcriptome of *P. brasiliensis*[66,67]. One study identified the *4-HPPD* gene to be required for *P. brasiliensis* conidia to convert to yeast [66]. They found that the *4-HPPD* gene expression was upregulated in the yeast form and that a biochemical inhibitor of this enzyme, nitisinone, inhibited mycelium conversion to yeast. *4-HPPD* (E.C. 1.13.1127) is an enzyme that converts 4-hydroxyphenylpyruvate to homogentisate that is involved in the synthesis of tyrosine, phenylalanine, and ubiqinone (KEGG, whttp://www.genome.jp/keg).

**Figure 5:**
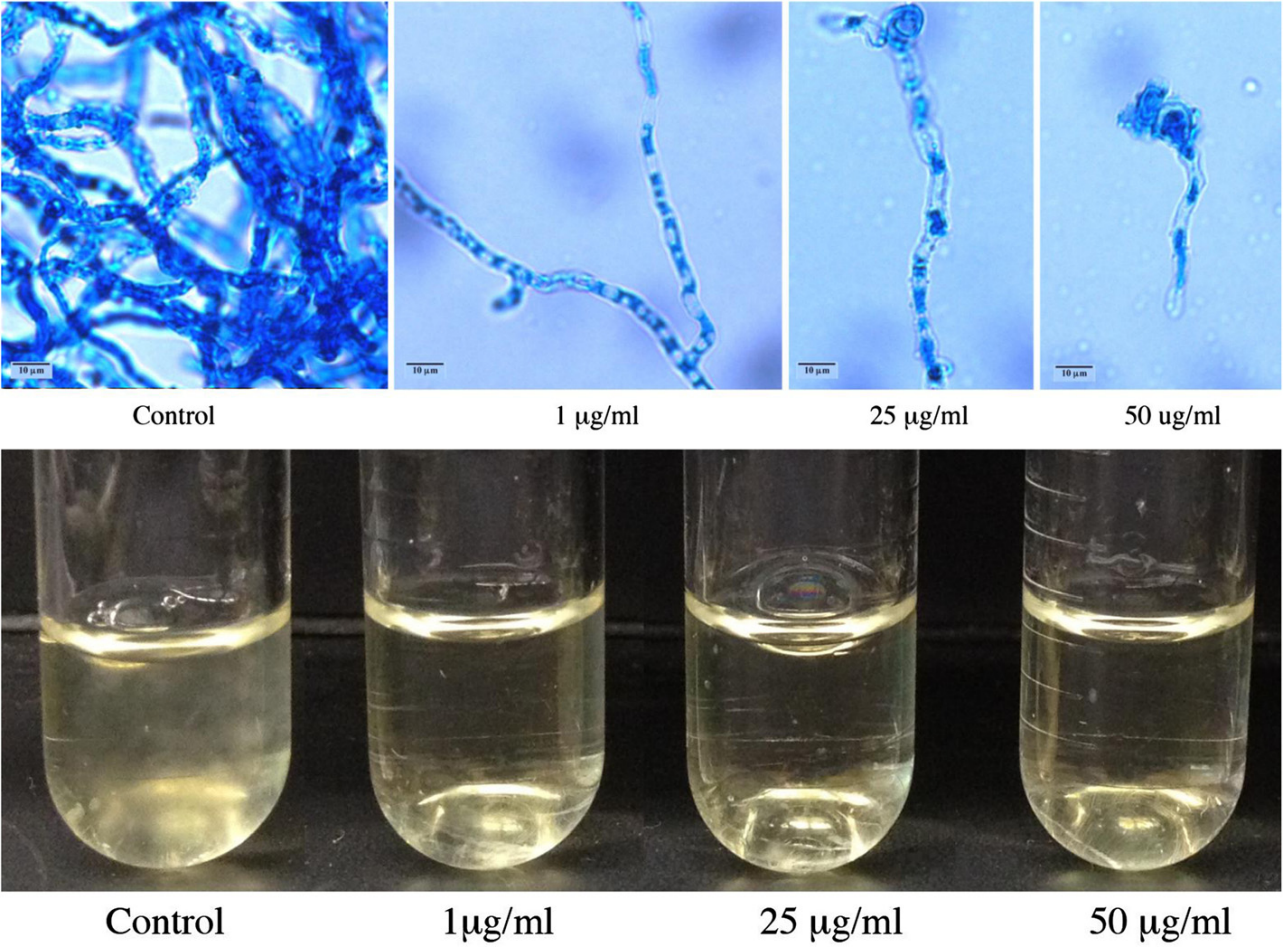
**Inhibition of *C. immitis *mycelial growth by nitisinone.** Photomicrographs showing (**A**) mycelial growth in the presence of nitisinone at doses of 1 μg/ml, 25 μg/ml and 50 μg/ml compared to the control; (**B**) mycelial growth as measured by turbidity in the indicated concentrations of nitisinone compared to the control.

There are two homologs of the *4-HPPD* in the *C. immitis* genome, which have significantly different sequences. One of these, CIMG_01466 was upregulated 5.27 fold in day 2 spherules and 3.80 fold in day 8 spherules. This gene was also found to be upregulated in spherules by Whiston et al. [13]. The other homolog, CIMG_01310, was downregulated −23.67 fold in day 2 spherules and −6.09 fold in day 8 spherules. The biggest difference in sequence is that CIMG_01466 has two substantial deletions compared to CIMG_01310. These deletions flank the highly conserved site that is predicted to contact the active site metal ion [68]. Furthermore, CIMG_01466 had substitutions in the predicted metal ion contact site, suggesting that it may not be an active enzyme. Nevertheless, we tested the effect of nitisinone on mycelial growth and mycelium to spherule conversion. We found that nitisinone inhibits mycelial growth at concentrations as low as 1 μg/ml (Figure 5). Surprisingly, there was no effect on mycelium to spherule conversion (data not shown). This is distinctly different from the results seem in *P. brasiliensis*. Our data suggests that *4-HPPD* enzyme activity is not required for mycelium to spherule conversion or the growth of spherules but it is important for mycelial growth.

## Conclusions

Conversion from the arthroconidia phase to the parasitic spherule phase in *C. immitis* requires major transcriptional reprogramming with 22% of the entire genome being differentially expressed between the two conditions. Further, gene expression within spherules is dynamic with 12% of the entire genome being differentially expressed as they mature from day 2 to day 8. It is evident from the transcriptional profile at day 2 compared to mycelia that differentiation of *C. immitis* is associated with the regulation of specific genes. For example, a number of genes were downregulated during mycelia to spherule conversion including transcriptional repressors (genes encoding zinc finger proteins), pleckstrin domain containing genes, and genes coding for proteins with SH3 signaling domains. Additionally, twenty-four protein kinase genes homologous to *S. cerevisiae* genes coding for sexual or meiotic function or mitosis or filamentous growth are downregulated and may play a role in arthroconidia differentiation to spherules. About 75% of the protein kinase genes return to mycelial levels of expression in 8 day spherules, suggesting they may be important in arthroconidia to spherule differentiation but not in spherule maturation. Some genes are persistently upregulated or downregulated in spherules at both time points. These include some genes that have previously been shown to be important for yeast development in *H. capsulatum* such as amylase gene *AMY-1*[62]. An enzyme such as extra-cellular superoxide dismutase (SOD3),that provides protection against oxidative stress was also upregulated in both day 2 and day 8 spherules [56]. In contrast to *Paracoccidioides brasiliensis*, where inhibition of the enzyme 4-HPPD inhibits conversion of the mold to the yeast form [66], inhibition of the enzyme 4-HPPD inhibits mycelial growth in *C. immitis* but has no effect in arthroconidia to spherule conversion in *C. immitis*. Arthroconidia to spherule conversion in *C. immitis* is a complex process requiring modulation of a large number of genes.

## Abbreviations

4-HPPD: 4-hydroxyphenylpyruvate dioxygenase; FC: Fold change; FDR: False discovery rate; GO: Gene ontology; GYE: Glucose yeast extract; ORF: Open reading frame; PFAM: Protein families.

## Competing interests

The authors declare that they have no competing interests.

## Authors’ contributions

SV grew the mycelia and spherules, did the inhibition experiments and prepared the RNA; AS performed most of the bioinformatic analysis; JF participated in writing the manuscript; JG did the bioinformatic analysis of protein kinases; TK supervised the experimental work and analyzed the bioinformatic results; CW supervised the bioinformatic analysis; all of the authors participated in writing the manuscript. All authors read and approved the final manuscript.

## Supplementary Material

Additional file 1: Table S1 GO terms associated with *C. immitis* locus tags. Click here for file

Additional file 2: Table S3 Classification of *C. immitis* protein kinases. Click here for file

Additional file 3: Figure S1 A dendrogram showing that unsupervised clustering using the expression of all genes on the microarray revealed that mycelia samples clustered distinctly from spherule samples. Furthermore, spherule samples formed two sub-clusters based on maturity. Click here for file

Additional file 4: Table S2 Genes identified as differentially expressed between the three experimental conditions: day 2 spherule vs mycelia, day 8 vs mycelia spherule and day 8 spherule vs day 2 spherule. Click here for file

Additional file 5: Figure S2 Two Venn diagrams revealing the partially overlapping pattern of gene expression between day 2 and day 8 spherules in this study and day 4 spherules in the Whiston et al. study [13]. Click here for file

Additional file 6: Figure S3 Hierarchical depiction of GO terms significantly over-represented in the set of genes that were differentially expressed with a fold change ≥ 2 or ≤ -2 between mycelia and day 8 spherules (A) or day 2 and day 8 spherules (B). The size of the node associated with each GO term is relative to the number of differentially expressed genes belonging to that term. The color scale indicates the level of significance associated with each node with red being the most significant. Click here for file

Additional file 7: Table S4 Genes overexpressed in day 2 spherules, day 8 spherules and in day 4 spherules as reported by Whiston et al. [13]. Click here for file
