# Supplementary figures and images for: Gene expression in human fungal pathogen Coccidioides immitis changes as arthroconidia differentiate into spherules and mature
Source: BMC Microbiol. 2013 May 28;13:121. doi: 10.1186/1471-2180-13-121 (PMC3693894; doi:10.1186/1471-2180-13-121)

## Slide 1
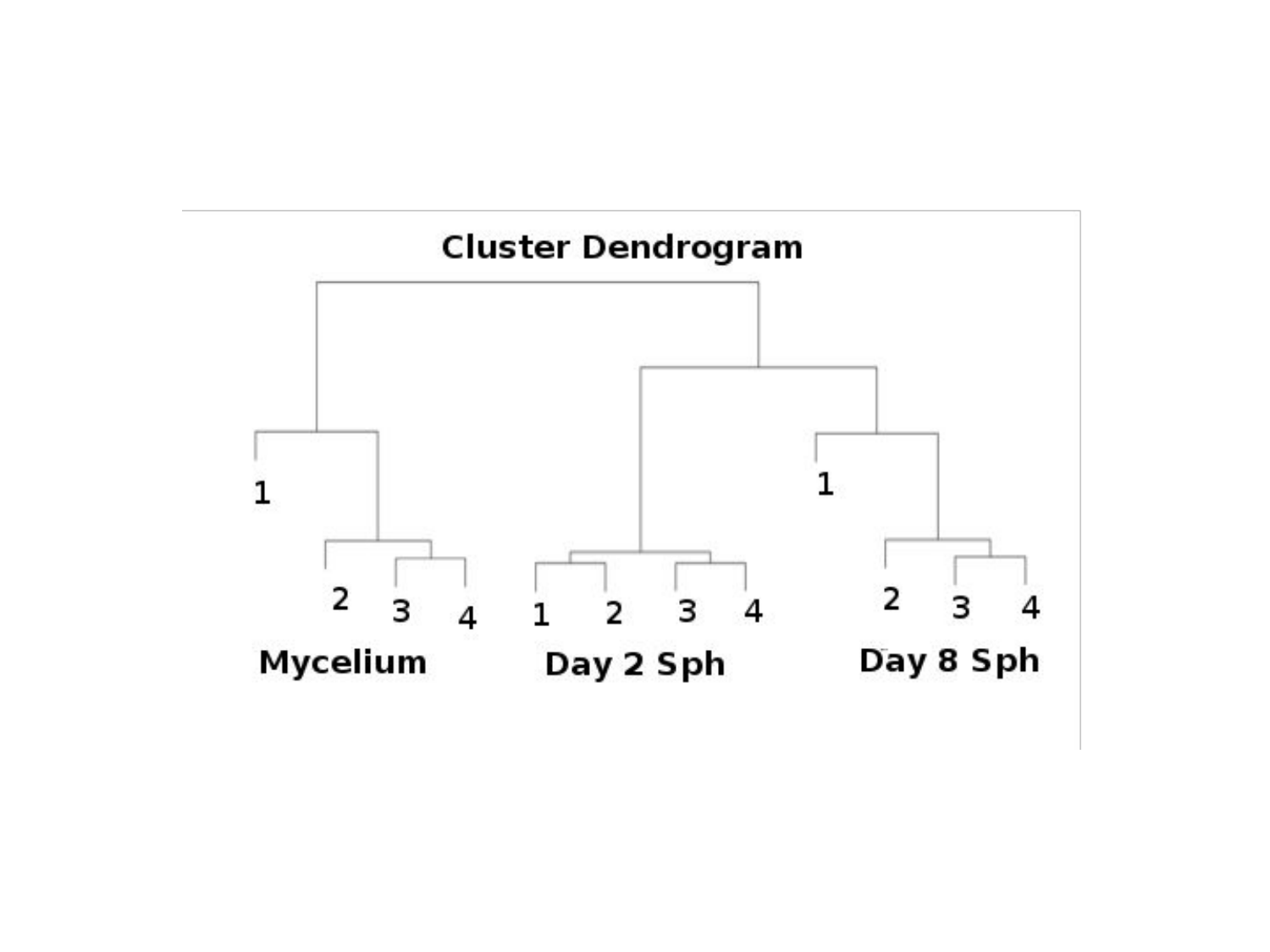

Supplement: Additional file 3: Figure S1 — A dendrogram showing that unsupervised clustering using the expression of all genes on the microarray revealed that mycelia samples clustered distinctly from spherule samples. Furthermore, spherule samples formed two sub-clusters based on maturity. [file 1471-2180-13-121-S3.pptx]

## Slide 1
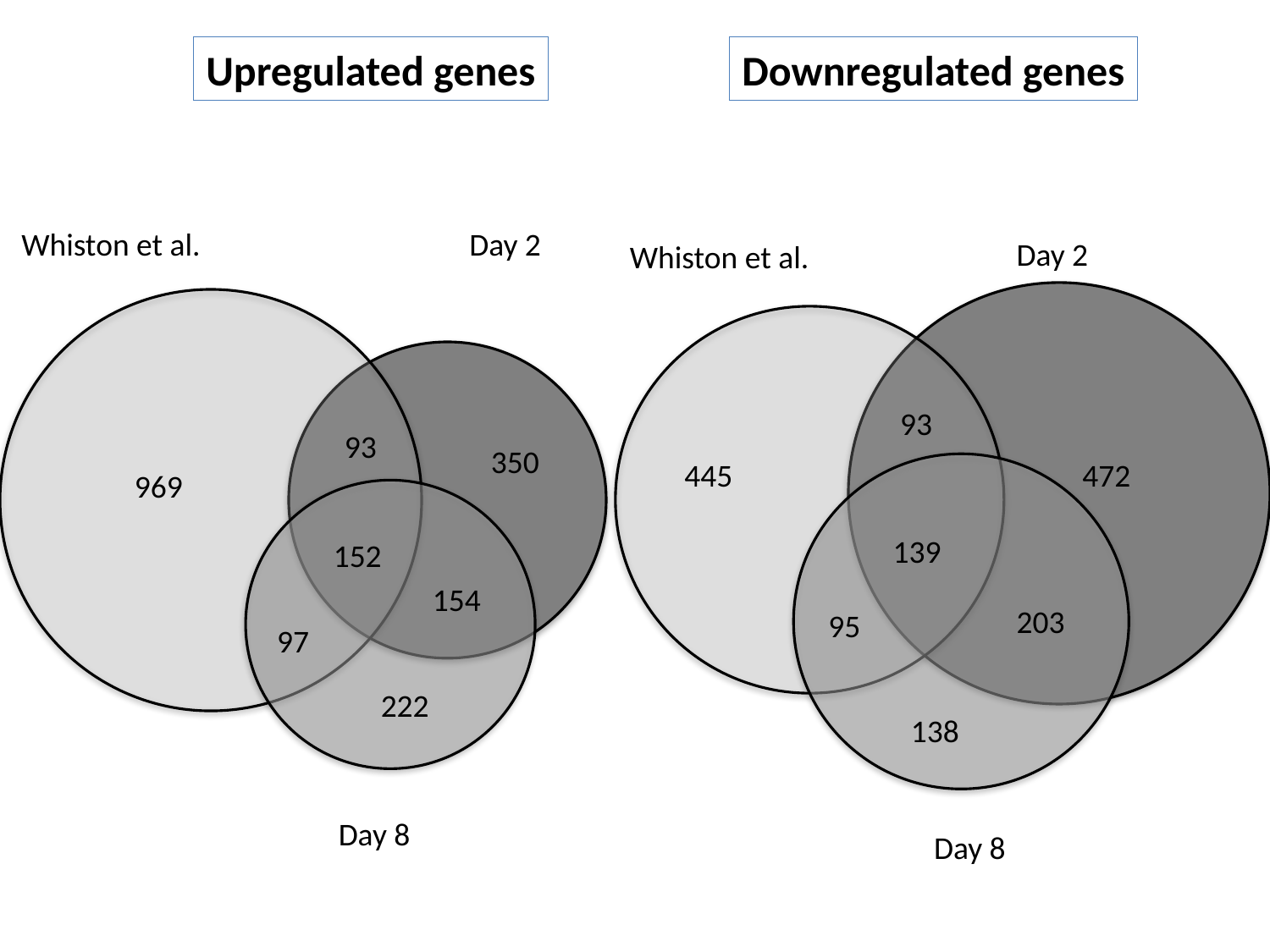

Upregulated genes
Downregulated genes
Whiston et al.
Day 2
93
350
969
152
154
97
222
Day 8
Day 2
Whiston et al.
93
445
472
139
203
95
138
Day 8

Supplement: Additional file 5: Figure S2 — Two Venn diagrams revealing the partially overlapping pattern of gene expression between day 2 and day 8 spherules in this study and day 4 spherules in the Whiston et al. study [13]. [file 1471-2180-13-121-S5.pptx]

## Slide 1
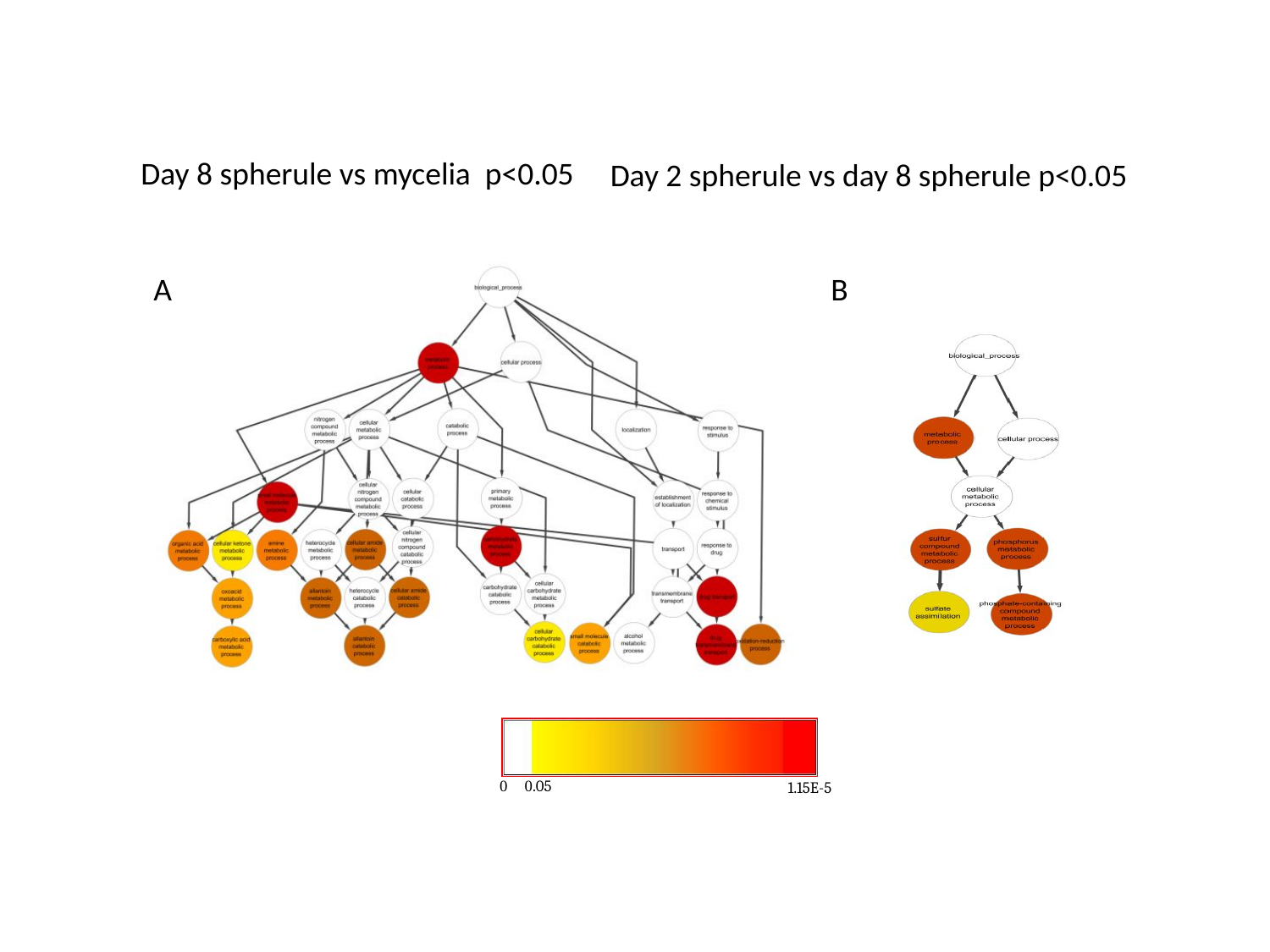

Day 8 spherule vs mycelia p<0.05
Day 2 spherule vs day 8 spherule p<0.05
A
B
1.15E-5
0.05
0

Supplement: Additional file 6: Figure S3 — Hierarchical depiction of GO terms significantly over-represented in the set of genes that were differentially expressed with a fold change ≥ 2 or ≤ -2 between mycelia and day 8 spherules (A) or day 2 and day 8 spherules (B). The size of the node associated with each GO term is relative to the number of differentially expressed genes belonging to that term. The color scale indicates the level of significance associated with each node with red being the most significant. [file 1471-2180-13-121-S6.pptx]
